# Sarcopenic obesity is associated with frailty among community-dwelling older adults: findings from the WCHAT study

**DOI:** 10.1186/s12877-022-03617-z

**Published:** 2022-11-16

**Authors:** Mei Yang, Meng Hu, Yan Zhang, Shuli Jia, Xuelian Sun, Wanyu Zhao, Meiling Ge, Birong Dong

**Affiliations:** 1 National Clinical Research Center for Geriatrics, West China Hospital, Sichuan University, GuoXueXiang 37, Chengdu, 610041 China; 2 National Clinical Research Center for Geriatrics and Department of General Practice, State Key Laboratory of Biotherapy, West China Hospital, Sichuan University, and Collaborative Innovation Center of Biotherapy, Chengdu, China; 3 Center of Gerontology and Geriatrics, West China Hospital, Sichuan University, GuoXueXiang 37, Chengdu, 610041 China

**Keywords:** Sarcopenic obesity, Frailty, Older adults

## Abstract

**Objective:**

Uncertainties remain regarding the relationship between sarcopenic obesity and frailty. This study aimed to explore the association of these two common geriatric syndromes among community-dwelling older adults.

**Methods:**

Baseline data from the West China Health and Aging Trend (WCHAT) study was used. Sarcopenia was assessed based on the criteria established by the Asian working group for sarcopenia. Body fat percentages above the 60th percentile specified by sex were classified as obesity. Sarcopenic obesity was defined as the concurrence of obesity and sarcopenia. Frailty was assessed by Fried criteria. Multinomial logistic regression was adopted to explore associations of sarcopenic obesity with frailty.

**Results:**

Overall, 2372 older adults (mean age 67.6 ± 5.9) were involved in this study. The prevalence of frailty and sarcopenic obesity was 6.2 and 6.28%, respectively. After adjusting for covariates, sarcopenic obesity was significantly associated with prefrailty (OR = 1.74, 95% CI = 1.15–2.64*, P* = 0.009) and frailty (OR = 4.42, 95% CI = 2.19–8.93*, P* < 0.001) compared to nonsarcopenia and nonobesity.

**Conclusions:**

Sarcopenic obesity was significantly correlated with prefrailty and frailty among older adults. Intervention for sarcopenic obesity may contribute to the prevention of incident frailty.

## Introduction

Frailty, characterized by increased susceptibility to stressors and decreased physiological reserves [1], is a multidimensional geriatric condition incorporating physical, psychological and social domains [2]. Frailty is a highly prevalent and health-threatening issue among older adults. Presently, several operational definitions of frailty have been proposed, among which the Fried phenotype [3] and the Frailty Index (FI) [4] are most frequently used. The prevalence of frailty differs significantly, ranging from 4 to 59% due to the lack of a unique definition [5]. The adverse outcomes of frailty are wide-ranging. Disability [6], falls [7], fractures, mortality [8], loneliness, depression [9], cognitive impairment, dementia [10] and hospitalization [11] are all reported to be correlated with frailty.

As a dynamic condition, prefrailty and frailty are believed to be reversible to some extent. Among numerous studies conducted on the management of frailty, the European SPRINTT project (sarcopenia and physical frailty in older people: multicomponent treatment strategies), a multicomponent strategy composed of nutritional and technological intervention, physical activity and educational counseling, has drawn our attention [12, 13]. It has been demonstrated that this multicomponent intervention could reduce the incidence of mobility disability [14] in physically frail or sarcopenic older adults. Despite the inspiring results of the project, identifying modifiable risk factors for frailty is still a priority for healthy aging.

Body composition changes with aging, and muscle mass usually decreases in conjunction with fat mass gain. The concurrence of excessive adiposity and low muscle mass is emerging as a major health problem termed ‘sarcopenic obesity’ [15]. Sarcopenic obesity consists of two components, namely, sarcopenia and obesity. Sarcopenia per se is closely related to frailty and has been regarded as a biological substrate of physical frailty [16]. Obesity has also been linked to frailty. A meta-analysis conducted by Yuan et al. revealed that both abdominal obesity (relative risk (RR) = 1.57, 95% confidence interval (CI) = 1.29–1.91) defined by waist circumference and general obesity defined by body mass (RR = 1.40, 95% CI = 1.17–1.67) could increase the risk of frailty [17]. In addition, an increased body fat percentage has also been reported to be associated with frailty (β = 0.97 ± 0.43, *p* = 0.03) [18]. Although no consensus has been reached regarding the diagnostic criteria of sarcopenic obesity, the hazardous effect of sarcopenic obesity should never be neglected.

Presently, associations of frailty with decreased muscle mass or increased body fat have been explored separately. However, little is known regarding the association between sarcopenic obesity and frailty. Whether sarcopenic obesity augments the deleterious effect of each condition remains unclear.

To bridge this gap, we conducted this study, which aimed to shed light on the prevalence of sarcopenic obesity, as well as the association between sarcopenic obesity and frailty in older adults.

## Methods

### Study design and sample selection

This was a retrospective, cross-sectional analysis of baseline data from the West China Health and Aging Trend (WCHAT) study. Details of the WCHAT study have been described elsewhere [19]. The WCHAT study was approved by the Ethics Committee of West China Hospital, Sichuan University (reference: 2017–445) and was carried out under the guidance of the Helsinki Declaration. This study was also registered at the Chinese Clinical Trial Registry (number ChiCTR1800018895; date of first registration 16/10/2018). Before enrollment, informed consent was obtained from each participant.

**Fig. 1:**
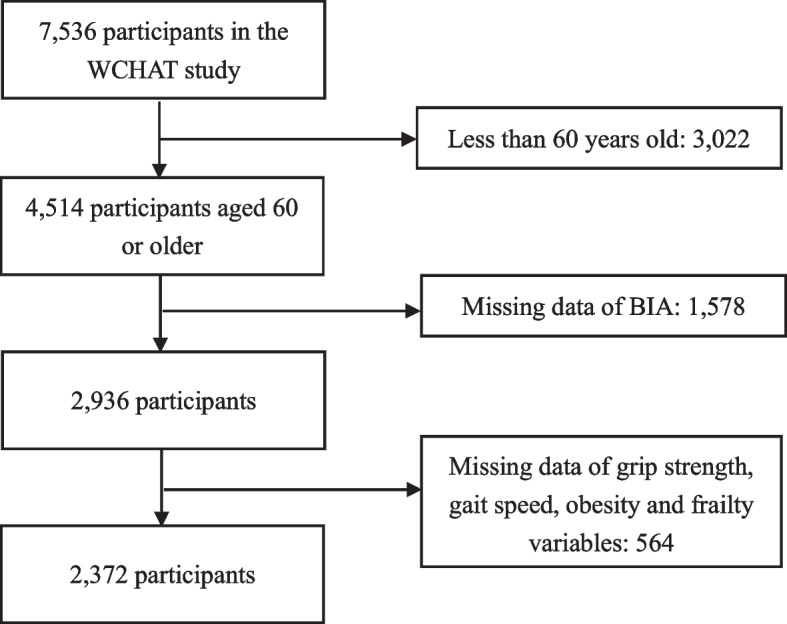
Flow chart of the participants

A total of 7536 participants were enrolled in the WCHAT study. Eventually, we included 2372 participants after excluding 3022 participants under 60 years old, 1578 with missing data for bioimpedance analysis, and 528 missing data for grip strength, gait speed, body fat percentage and frailty phenotype (Fig. 1).

### Assessment of frailty

Frailty was assessed based on the modified Fried phenotype [3]. Five components were used to define frailty, including shrinking, weakness, exhaustion and slowness. Participants were divided into 3 groups according to the number of components involved (0 component for robust, 1 or 2 components for prefrailty and 3 or more components for frailty). The details of each component are described below.

1. Shrinking: shrinking was defined as an unintentional weight loss of more than 4.5 kg during the past year or a body mass index (BMI) < 18.5 kg/m^2^.
2. Weakness: Weakness was defined as grip strength of the dominant hand in the lowest quintile of the population distribution, adjusted for sex and body mass index (BMI).
3. Exhaustion: meeting any one of the criteria below was considered exhaustion. (1) I felt extremely fatigued for the majority of the time; (2) I felt extremely weak for the majority of the time; (3) A self-reported energy score of three or less was reported when a score of ten represents the condition with the greatest power.
4. Slowness: 4-m walking time in the lowest quintile of the population distribution, adjusted for sex and height.
5. Low physical activity: Sex-adjusted kilocalories in the lowest quintile based on a validated China Leisure Time Physical Activity Questionnaire (CLTPAQ) [20]

### Assessment of sarcopenia, obesity and sarcopenic obesity

Sarcopenia was assessed based on the criteria established by the Asian Working Group for Sarcopenia (AWGS) in 2019 [21]. The appendicular skeletal muscle index (SMI) was used as an indicator for muscle mass. SMI and body fat percentage were calculated with a bioimpedance analyzer (InBody 770, Biospace, Korea). The cutoffs for low muscle mass were 7.0 kg/m^2^ and 5.7 kg/m^2^ in men and women, respectively. Dynamometers (EH101; Camry, Zhongshan, China) were used to measure grip strength. The cutoffs for low grip strength were 28 kg for males and 18 kg for females. A cutoff of 1.0 m/s for gait speed was used to estimate physical function. Body fat percentages exceeding the 60th percentile specified by sex were classified as obesity [22]. Concurrence of obesity and sarcopenia was defined as sarcopenic obesity [23].

### Covariates

Information including age, sex, education level (illiteracy/primary school/secondary school or above), ethnicities (Han/Yi/Tibetan/Qiang/other ethnic minorities), smoking history, alcohol history, marital status (married/single), and number of chronic diseases (0/1/≥ 2) were collected via face-to-face interviews. Nutrition status was categorized using the Mini Nutrition Assessment-Short Form (MNA-SF) scale (0 ~ 11 scores as malnutrition risk; 12 ~ 14 scores as well nourished) [24].

### Statistical analysis

We conducted the analyses with Stata software, version 14.0 (Stata Corp, College Station, TX, USA). Continuous data are presented as the means ± standard deviations (SD) or medians and interquartile range (IQR), while categorical variables are presented as counts (percentages). Group differences were tested by ANOVA or Kruskal-Wallis for normally distributed or skewed continuous variables and the chi square test for categorical variables, respectively. Multinomial logistic regression was adopted to explore the associations of frailty with sarcopenic obesity. Variables such as age, sex, ethnicity, education level, marital status, smoking history, drinking history, number of chronic diseases, and risk of malnutrition were included in the adjusted model. Each statistical test was two-sided, and *P* < 0.05 was set as the significance level.

## Results

In total, 2372 participants (mean age 67.6 ± 5.9 years; 60.24% female) were included in this analysis. The prevalence rates of obesity alone, sarcopenia alone and sarcopenic obesity were 33.05, 23.31 and 6.28%, respectively. The percentages of prefrailty and frailty were 46.96 and 6.2%, respectively.

Table 1 presents the characteristics of the participants according to sarcopenia and obesity status. Significant differences regarding age, sex, ethnicities, education level, smoking history, marital status, number of chronic diseases, nutritional status and frailty status, were observed among the 4 groups. Participants with sarcopenia alone or sarcopenic obesity were older than those in the obesity alone group or the nonobese and nonsarcopenia group.

**Table 1:** Characteristics of participants according to sarcopenia and obesity status

	Neither sarcopenia nor obesity *n* = 886	Sarcopenia alone *n* = 553	Obesity alone *n* = 784	Sarcopenic obesity *n* = 149	*P* value
Age, y*	65 (62–70)	70 (65–75)	66 (63–70)	69 (65–75)	< 0.001
Female, %	548 (61.9)	317 (57.3)	496 (63.3)	68 (45.6)	< 0.001
Education level, %					0.006
Illiterate	306 (36.0)	210 (40.0)	272 (36.5)	46 (32.6)	
Primary school	337 (39.6)	208 (39.6)	281 (37.7)	43 (30.5)	
Secondary school and above	207 (24.4)	107 (20.4)	193 (25.9)	52 (36.9)	
Ethnicity, %					< 0.001
Han	396 (44.7)	302 (54.6)	282 (36.0)	67 (45.0)	
Qiang	286 (32.3)	87 (15.7)	250 (31.9)	32 (21.5)	
Tibetan	154 (17.4)	102 (18.4)	213 (27.2)	44 (29.5)	
Yi	43 (4.9)	55 (9.9)	24 (3.1)	4 (2.7)	
others	7 (0.8)	7 (1.3)	15 (1.9)	2 (1.3)	
Marital status, %					0.036
Married	690 (81.2)	397 (75.6)	609 (81.6)	110 (78.0)	
Unmarried/widowed/divorced	160 (18.8)	128 (24.4)	137 (18.4)	31 (22.0)	
History of smoke, %	157 (18.6)	135 (26.0)	93 (12.6)	37 (26.2)	< 0.001
History of alcohol, %	232 (27.5)	142 (27.3)	194 (26.1)	38 (27.0)	0.94
Number of chronic diseases, %					0.014
0	486 (57.2)	306 (58.6)	365 (49.1)	79 (56.0)	
1	203 (23.9)	119 (22.8)	221 (29.7)	33 (23.4)	
> = 2	161 (18.9)	97 (18.6)	157 (21.1)	29 (20.6)	
Nutritional status, %					< 0.001
Well nourished	702 (83.0)	278 (53.6)	659 (89.2)	115 (81.6)	
Risk of malnutrition	144 (17.0)	241 (46.4)	80 (10.8)	26 (18.4)	
Frailty status, %					< 0.001
Robust	468 (52.8)	201 (36.3)	394 (50.3)	48 (32.2)	
Pre-frailty	386 (43.6)	295 (53.3)	355 (45.3)	78 (52.3)	
Frailty	32 (3.6)	57 (10.3)	35 (4.5)	23 (15.4)	

* Data are presented as the medians and interquartile range (IQR); Significance was accepted at *P* < .05

Table 2 shows the results of logistic regression about the association of frailty with sarcopenic obesity. We found that in the unadjusted model, sarcopenic obesity and sarcopenia alone were significantly related to prefrailty and frailty compared with the nonobesity and nonsarcopenia groups, whereas obesity alone was not. The odds ratios for prefrailty were 1.77 (95% CI = 1.42–2.22, *P* < 0.001) in the sarcopenia alone group and 1.97 (95% CI = 1.34–2.89, *P* < 0.001) in the sarcopenic obesity group. In addition, the odds ratios for frailty were 4.14 (95% CI = 2.60–6.59, *P* < 0.001) in the sarcopenia alone group and 7.00 (95% CI = 3.79–12.93*, P* < 0.001) in the sarcopenic obesity group. However, after adjustment for confounders, only sarcopenic obesity was independently associated with prefrailty and frailty. The respective odds ratios for prefrailty and frailty were 1.74 (95% CI = 1.15–2.64*, P =* 0.009) and 4.42 (95% CI = 2.19–8.93*, P* < 0.001), respectively.

**Table 2:** Association between sarcopenic obesity and frailty

	Pre-frailty vs. Robust		Frailty vs. Robust	
	OR [95%CI]	*P* value	OR [95%CI]	*P* value
Unadjusted model				
Non-sarcopenia and Nonobesity	Ref.	NA	Ref.	NA
Sarcopenia alone	1.77 [1.42, 2.22]	< 0.001	4.14 [2.60, 6.59]	< 0.001
Obesity alone	1.09 [0.89, 1.33]	0.379	1.29 [0.78, 2.13]	0.303
Sarcopenic obesity	1.97 [1.34, 2.89]	< 0.001	7.00 [3.79, 12.93]	< 0.001
Adjusted model ^a^				
Non-sarcopenia and Nonobesity	Ref.	NA	Ref.	NA
Sarcopenia alone	1.21 [0.93, 1.56]	0.146	1.42 [0.83, 2.44]	0.193
Obesity alone	1.11 [0.89, 1.37]	0.337	1.50 [0.87, 2.57]	0.139
Sarcopenic obesity	1.74 [1.15, 2.64]	0.009	4.42 [2.19, 8.93]	< 0.001

*OR *Odds Ratio, *CI *Confidence Interval, *Ref*. Reference, *NA *Non-applicable

Model ^a^: adjusted for age, gender, education, ethnicity, marital status, history of smoking, history of drinking, number of chronic diseases, risk of malnutrition

We further explored sex and age differences regarding the association of frailty with sarcopenic obesity in a fully adjusted model. After stratification by sex, the association of frailty with sarcopenic obesity remained significant. The respective odds ratios were 7.14 (95% CI = 2.19–23.97*, P* = 0.001) and 4.18 (95% CI = 1.63–10.72*, P* = 0.003) for males and females, respectively. However, an association of sarcopenic obesity with prefrailty was observed only in males (OR = 2.00, 95% CI = 1.12–3.57*, P* = 0.018) and not in females (OR = 1.40, 95% CI = 0.76–2.61*, P* = 0.276) (Table 3). Regarding different age groups, sarcopenic obesity was demonstrated to be significantly associated with prefrailty (OR = 2.84, 95% CI = 1.32–6.13*, P* = 0.007) and frailty (OR = 6.86, 95% CI = 2.52–18.64*, P* < 0.001) in participants aged 70 years and over. However, in participants aged 60–69 years, sarcopenic obesity was only related to frailty (OR = 3.79, 95% CI = 1.16–12.42*, P* = 0.027) rather than prefrailty (OR = 1.45, 95% CI = 0.86–2.44*, P* = 0.153) (Table 4).

**Table 3:** Association between sarcopenic obesity and frailty stratified by sex in adjusted model

	Pre-frailty vs. Robust		Frailty vs. Robust	
	OR [95%CI]	*P* value	OR [95%CI]	*P* value
Men				
Non-sarcopenia and Nonobesity	Ref.	NA	Ref.	NA
Sarcopenia alone	1.30 [0.87, 1.95]	0.197	3.22 [1.15, 8.97]	0.025
Obesity alone	0.96 [0.67, 1.38]	0.673	3.06 [1.09, 8.56]	0.033
Sarcopenic obesity	2.00 [1.12, 3.57]	0.018	7.14 [2.13, 23.97]	0.001
Women				
Non-sarcopenia and Nonobesity	Ref.	NA	Ref.	NA
Sarcopenia alone	1.14 [0.81, 1.60]	0.435	1.00 [0.51, 1.95]	0.996
Obesity alone	1.17 [0.90, 1.54]	0.229	1.05 [0.53, 2.08]	0.880
Sarcopenic obesity	1.40 [0.76, 2.61]	0.276	4.18 [1.63, 10.72]	0.003

*OR *Odds Ratio, *CI *Confidence Interval, *Ref*. Reference, *NA *Non-applicable

**Table 4:** Association between sarcopenic obesity and frailty stratified by age in adjusted model

	Pre-frailty vs. Robust		Frailty vs. Robust	
	OR [95%CI]	*P* value	OR [95%CI]	*P* value
60–69 years				
Non-sarcopenia and Nonobesity	Ref.	NA	Ref.	NA
Sarcopenia alone	1.07 [0.77, 1.49]	0.650	1.49 [0.63, 3.53]	0.355
Obesity alone	1.13 [0.88, 1.44]	0.326	1.93 [0.88, 4.21]	0.098
Sarcopenic obesity	1.45 [0.86, 2.44]	0.153	3.79 [1.16, 12.42]	0.027
≥70 years				
Non-sarcopenia and Nonobesity	Ref.	NA	Ref.	NA
Sarcopenia alone	1.59 [1.02, 2.48]	0.037	1.53 [0.74, 3.15]	0.245
Obesity alone	1.19 [0.77, 1.85]	0.420	1.31 [0.60, 2.87]	0.490
Sarcopenic obesity	2.84 [1.32, 6.13]	0.007	6.86 [2.52,18.64]	< 0.001

*OR *Odds Ratio, *CI *Confidence Interval, *Ref*. Reference, *NA *Non-applicable

## Discussion

This study first examined the association of sarcopenic obesity with frailty among community-dwelling older adults in western China. Our results revealed that individuals with sarcopenic obesity had 1.74 times and 4.42 times increased risks for prefrailty and frailty, respectively.

In our study, the prevalence of frailty was 6.2%, which was in accordance with previously reported data in the Chinese community [25]. The prevalence of frailty was 15.4, 10.3 and 4.5% in the sarcopenic obesity group, sarcopenia alone group and obesity alone group, respectively. Our study indicated that sarcopenic obesity significantly increased the risks of prefrailty and frailty. These findings were supported by Hirani and colleagues, who reported that men with sarcopenic obesity were more prone to frailty, with an odds ratio of 2.0 (95% CI = 1.42–2.82) [26]. In addition, Saitoh et al. also reported that sarcopenic obesity could increase the risk of frailty with an odds ratio of 4.518 (95% CI = 1.218–16.752, *P* = 0.024) in patients undergoing hemodialysis [27].

The results of our study demonstrated that neither sarcopenia alone nor obesity alone fully captured the vital link to frailty, which was partially different from previous studies. This discrepancy possibly resulted from ethnic differences as well as variance in diagnostic criteria for obesity, sarcopenia and frailty. Meanwhile, decreased muscle mass along with increased body fat may not have been taken into consideration simultaneously in these studies, which may obscure the role of obesity in sarcopenia and vice versa. After age stratification, a significant association between sarcopenic obesity and prefrailty was found only in individuals aged 70 and over. This is possibly because of the dynamic nature and complex diagnostic components of prefrailty as well as the dominant role of aging in the development of prefrailty. Presently, most studies have focused on associations of frailty with obesity or sarcopenia separately. For example, Falsarella et al. reported that a significant difference in body fat percentage was observed between nonfrail and frail individuals [28]. In addition, sarcopenia components, including decreased muscle mass [29], decreased grip strength [30] and decreased gait speed [31], have also been reported to be related to frailty. However, a recent study disclosed that sarcopenia predicted frailty with a high specificity (> 97%) but a low sensitivity (< 10%) [32]. Considering that an aging-related increase in fat mass is always coupled with a decrease in muscle mass, the combined effect of sarcopenia and obesity could be more predictive.

The mechanism linking sarcopenic obesity with frailty remains unclear. Here, we provide some insights into the similarities between the two diseases. Biological factors contributing to the development of frailty overlap significantly with those described for sarcopenic obesity. Weakness is reported to present as an initial sign of frailty [33]. Fat infiltration including intermuscular adipose tissue (IMAT) and intramyocellular lipids (IMCLs) in skeletal muscle is reported to be associated with muscle weakness [34]. On the one hand, IMCLs could impair mitochondrial function, reduce lipid β-oxidation and enhance reactive oxygen species (ROS) production. Increased ROS could activate stress pathways such as c-jun N-terminal kinase(JNK), I*κ*B kinase (IKK), and p38-mitogen-activated protein kinase (p38-MAPK), which compromise the function of muscle protein [35]. On the other hand, inflammation is regarded as the common pathogenesis of sarcopenia, sarcopenic obesity and frailty [36, 37]. IMAT is reported to enhance whole-body inflammation as well as induce local inflammation and insulin resistance by releasing a number of proinflammatory cytokines, which could impair muscle function [38, 39]. A meta-analysis showed that inflammatory markers such as C reactive protein (CRP), interleukin-6 (IL-6), and tumor necrosis factor α (TNF-α) are associated with lower grip strength (CRP; *r* = − 0.10, *p* < 0.001, IL-6; *r* = − 0.13, *p* < 0.001, TNFα; *r* = − 0.08, *p* < 0.00) [40]. It has been reported that CRP can inhibit Akt phosphorylation, downregulate the mammalian target of rapamycin (mTORC1) pathway, and inhibit the synthesis of muscle fibrin [41]. In addition, IL-6 may exert detrimental effects by upregulating ubiquitin, E3 ligase, and proteasome activity as well as by activating the nuclear factor kappa-B (NF-κB) pathway. Moreover, TNF-α can not only downregulate the expression of myogenic genes but also upregulate atrophy-associated genes such as muscle atrophy F-box (MuRF1) and muscle ring-finger protein 1 (MAFbx) by activating ubiquitin proteasome signaling and the NF-κB pathway [42].

The strength of our study was that we first investigated the association of sarcopenic obesity with frailty based on a relatively large sample size in western China. Nevertheless, there inevitably existed some limitations. First, the causal association of sarcopenic obesity with frailty cannot be confirmed by a cross-sectional study. In the future, longitudinal studies are needed to verify their relationship. Second, studies on sarcopenic obesity are hindered by the absence of a unified definition to a great extent. Third, the retrospective nature of the study may have introduced bias. Finally, there were other potential confounders we failed to address, such as dietary preference and medical conditions (chronic diseases, drug use and hospitalization et.al).

## Conclusion

Our findings indicated that sarcopenic obesity was significantly associated with frailty among older adults. As sarcopenic obesity is a hazardous but potentially modifiable condition, intervention for sarcopenic obesity may contribute to the prevention of incident frailty.

## Data Availability

The data that support the findings of this study are available from the National Clinical Research Center for Geriatrics, West China Hospital, but restrictions apply to the availability of these data, which were used under license for the current study and are not publicly available. Data are, however, available from the corresponding author upon reasonable request and with permission of the National Clinical Research Center for Geriatrics, West China Hospital.

## Abbreviations

SPRINTT: Sarcopenia and physical frailty in older people: multicomponent treatment strategies
RR: Relative risk
OR: Odds ratio
CI: Confidence interval
WCHAT: West China Health and Aging Trend study
BMI: Body mass index
CLTPAQ: China leisure time physical activity questionnaire
AWGS: Asian Working Group for Sarcopenia
SMI: Appendicular skeletal muscle index
MNA-SF: Mini Nutrition Assessment-Short Form
SD: Standard deviation
IQR: Interquartile range
IMAT: Intermuscular adipose tissue
IMCLs: Intramyocellular lipids
ROS: Reactive oxygen species
JNK: c-jun N-terminal kinase
IKK: I*κ*B kinase
p38-MAPK: p38-mitogen-activated protein kinase
CRP: C reactive protein
IL-6: Interleukin-6
TNF-α: Tumor necrosis factor α
mTORC1: Mammalian target of rapamycin
NF-κB: Nuclear factor kappa-B
MuRF1: Muscle atrophy F-box
MAFbx: Muscle ring-finger protein 1
NA: Nonapplicable
